# Niobium Nitride Nb_4_N_5_ as a New High‐Performance Electrode Material for Supercapacitors

**DOI:** 10.1002/advs.201500126

**Published:** 2015-07-15

**Authors:** Houlei Cui, Guilian Zhu, Xiangye Liu, Fengxin Liu, Yian Xie, Chongyin Yang, Tianquan Lin, Hui Gu, Fuqiang Huang

**Affiliations:** ^1^ CAS Key Laboratory of Materials for Energy Conversion and State Key Laboratory of High Performance Ceramics and Superfine MicrostructureShanghai Institute of CeramicsChinese Academy of SciencesShanghai200050China; ^2^ Beijing National Laboratory for Molecular SciencesCollege of Chemistry and Molecular EngineeringPeking UniversityBeijing100871China

**Keywords:** areal capacitance, cycling stability, Nb_4_N_5_ nanochannels, pseudocapacitance, supercapacitors

## Abstract

Supercapacitors suffer either from low capacitance for carbon or derivate electrodes or from poor electrical conductivity and electrochemical stability for metal oxide or conducting polymer electrodes. Transition metal nitrides possess fair electrical conductivity but superior chemical stability, which may be desirable candidates for supercapacitors. Herein, niobium nitride, Nb_4_N_5_, is explored to be an excellent capacitive material for the first time. An areal capacitance of 225.8 mF cm^−2^, with a reasonable rate capability (60.8% retention from 0.5 to 10 mA cm^−2^) and cycling stability (70.9% retention after 2000 cycles), is achieved in Nb_4_N_5_ nanochannels electrode with prominent electrical conductivity and electrochemical activity. Faradaic pseudocapacitance is confirmed by the mechanistic studies, deriving from the proton incorporation/chemisorption reaction owing to the copious +5 valence Nb ions in Nb_4_N_5_. Moreover, this Nb_4_N_5_ nanochannels electrode with an ultrathin carbon coating exhibits nearly 100% capacitance retention after 2000 CV cycles, which is an excellent cycling stability for metal nitride materials. Thus, the Nb_4_N_5_ nanochannels are qualified for a candidate for supercapacitors and other energy storage applications.

## 1 Introduction

As a promising energy storage device for portable electronics and electric vehicles in the future, supercapacitors (also called ultracapacitors or electrochemical capacitors), may fill the gap between secondary batteries and traditional capacitors.^1^, ^2^, ^3^, ^4^ Supercapacitors possess the advantages of simple construction mode, high charge/discharge rate, long cycle life, and high power density over secondary batteries,^4^, ^5^, ^6^, ^7^ with higher energy density than traditional capacitors.^1^, ^8^ Generally, some metal oxides like RuO_2_, MnO_2_, and NiO, carbon materials, and conducting polymers are the most common electrode materials for supercapacitors and have been widely investigated.^2^, ^3^, ^4^, ^8^ In addition, transition metal nitrides with low cost, excellent electrochemical property, high molar density, and superior chemical stability, are also promising choices for outstanding electrode materials.^9^, ^10^ Different transition metal nitrides, such as TiN, VN, WN, Mo_2_N, etc., have been studied as electrode materials for supercapacitor.^11^, ^12^, ^13^, ^14^, ^15^, ^16^ Among them, VN was reported to deliver a relatively high capacitance of 1340 F g^−1^ at a scan rate of 2 mV s^−1^,^11^ in contrast to the relatively lower capacitances achieved by WN (30 F g^−1^)^13^ and Mo_2_N (172 F g^−1^).^17^ TiN with various nanostructures like nanowires, nanotubes, and mesoporous microspheres were prepared to serve as promising electrodes for high energy supercapacitors.^15^, ^18^, ^19^, ^20^ More importantly, nanostructured TiN can also be used as a promising electronic conducting framework for high‐performance electrodes, due to its superior electrical conductivity and mechanical stability. By combining with other electroactive materials, a variety of TiN‐based composite electrodes with good performance were fabricated, such as TiN‐VN core–shell fibers (247.5 F g^−1^),^21^ MnO_2_/TiN nanotube coaxial arrays (681 F g^−1^),^22^ coaxial polyaniline/TiN/polyaniline nanotube arrays (242 mF cm^−2^),^23^ etc. Despite the multitudinous exploration of metal nitrides for supercapacitors, there has not been much work reported on the study of niobium nitride electrodes. To the best of our knowledge, Choi was the only one who prepared nanocrystalline niobium nitride powders and simply investigate their supercapacitor performance.^14^ The highest specific capacitance of 73 F g^−1^ was achieved for NbN crystallites at a scan rate of 2 mV s^−1^ in 1 m KOH electrolyte, with a quite narrow electrochemical potential window of 0.3 V.

Transition metal nitrides should gain capacitance from electrochemical storage of electricity energy achieved by redox reactions with specifically adsorbed ions from the electrolyte, i.e., protons in an aqueous electrolyte, accompanied with electrons transfer, like the case of MnO_2_ and RuO_2_. Generally, among transition metal materials, only those with high‐valence states could yield Faradaic pseudocapacitance. For instance, high specific capacitance of nano VN was attributed to the active material of a few nanometer oxidized surface comprising high‐valence V ions,^11^, ^16^, ^24^ so did nanostructured TiN. The corresponding bulk oxides possess relatively low specific capacitances, due to their highly insulating nature. In contrast, these very conductive nitrides as substrates favor electron transport back and forth to the oxidized surface layers, which enhance their specific capacitances. At this point, TiN, VN, NbN, and WN with relatively lower +3 oxidation state are all not good candidates for electrode materials. Thus, transition metal nitrides with high‐valence states deserve further exploration toward high‐performance electrochemical capacitors. Niobium nitrides including Nb_2_N, Nb_4_N_3_, NbN, etc., are well known as superconductors.^25^ Nitrogen‐rich phases Nb_4_N_5_ and Nb_5_N_6_ have also been presented in Nb—N system,^25^ which comprise high‐valence (+5) Nb ions, and thus may be promising candidates for electrochemical capacitors.

Since the capacitance of supercapacitors heavily depends on the specific surface area of the electrode materials, nanostructured materials with extremely large effective area hold great promise in the progress of supercapacitor technologies.^27^ Materials with nanotube/nanopore structure have attracted particular attention for supercapacitor electrodes,^5^, ^22^, ^28^, ^29^, ^30^, ^31^ for they could offer good accessibility for electrolyte and a short distance of ion diffusion or mass transport due to their unique dimensional structure and high surface/volume ratio. As a powerful synthesis method of nanostructured materials, anodization technique allows the manufacture of highly ordered and self‐organized nanoporous or nanotubular oxide structures with a wide variety of metals at relatively mild synthesis conditions, such as Ti, W, Zr, Nb, and Ta.^32^, ^33^, ^34^, ^35^, ^36^ Recently, highly ordered Nb_2_O_5_ nanochannels with different thicknesses have been prepared by anodizing Nb foil in a glycerol based electrolyte at elevated temperatures.^37^

For the capacitive performance of supercapacitor electrodes using nanostructured transition metal nitrides, cycling stability is likely to be one concern, because metal nitrides electrodes commonly suffer from severe capacitance decay in the long‐term process, due to irreversible electrochemical oxidation.^11^, ^12^, ^15^, ^16^, ^20^ Thus, their potential applications in high‐performance supercapacitors are seriously restricted by the rather poor cycling stability. Coating of a stable carbon layer has been a remedy to improve the cyclic performance of supercapacitor electrodes.^20^, ^38^, ^39^ Among many carbon sources for carbon coating like glucose, citric acid, and toluene, dopamine comprising high concentrations of catechol and amine functional groups, is a superior and flexible agent for surface coating of inorganic nanoparticles.^40^, ^41^

Herein, we report highly ordered Nb_4_N_5_ nanochannels prepared by anodizing of Nb foil and subsequent annealing in ammonia atmosphere. An areal capacitance of 225.8 mF cm^−2^ at a current density of 0.5 mA cm^−2^ is achieved in 1 m H_2_SO_4_ electrolyte, among the best level of metal nitrides nanostructured film electrodes. Reasonable electrochemical stability is confirmed by the 70.9% retention of initial capacitance after 2000 CV cycles. Moreover, this Nb_4_N_5_ nanochannels electrode with an ultrathin carbon coating exhibits nearly 100% capacitance retention after 2000 CV cycles, which is an excellent cycling stability for metal nitride materials.

## 2 Results and Discussion

### 2.1 Preparation and Characterization of Nb_4_N_5_ Nanochannels

**Figure 1:**
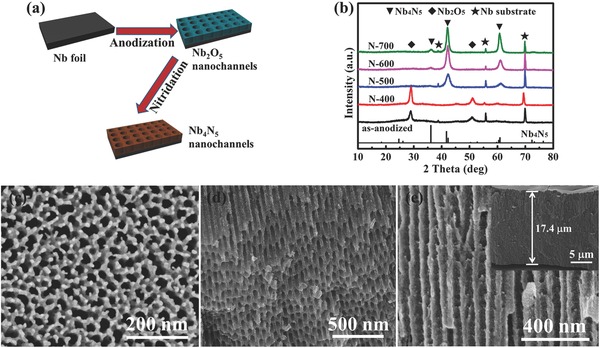
a) Schematic diagram illustrating the fabrication of Nb_4_N_5_ nanochannels. b) XRD patterns of as‐anodized sample and samples nitrided at different temperature. c) Typical top view, d) oblique cross‐section view, and e) side view FE‐SEM images of 700 °C nitrided Nb_4_N_5_ nanochannels.

The preparation of highly ordered Nb_4_N_5_ nanochannels mainly comprises two procedures, as schematically illustrated in **Figure** **1**a. First, the cleaned and chemically polished Nb foil was anodically oxidized for 15 min at 20 V, in an anhydrous glycerol solution containing 10 wt% K_2_HPO_4_ at 180 °C, to fabricate highly ordered Nb_2_O_5_ nanochannels, in reference to a previous report.^37^ Different from the generally reported as‐anodized amorphous TiO_2_ nanotubes,^42^ the as‐prepared Nb_2_O_5_ nanochannels possess a moderate crystallinity (TT‐Nb_2_O_5_, pseudohexagonal), as the X‐ray diffraction (XRD) pattern shows in Figure 1b. The relatively high operating temperature of 180 °C may cause the crystallization of Nb_2_O_5_ nanochannels immediately after their formation. However, the relatively weak intensity and large width of the diffraction peaks suggest the incomplete crystallization of the as‐prepared Nb_2_O_5_ nanochannels, namely, amorphous Nb_2_O_5_ also exists in the sample. Second, the as‐anodized Nb_2_O_5_ nanochannels were directly nitrided in NH_3_ atmosphere at a temperature range from 400 to 700 °C for 4 h. Figure 1b shows that the sample nitrided at 400 °C (defined as N‐400) possesses a similar XRD pattern with the as‐anodized sample, and delivers relatively higher peak intensity. Except for Nb_2_O_5_ phase and Nb substrate, there is no other phase appearing in the pattern, indicating only further crystallization, rather than thermal nitridation reaction, is the dominate process in the as‐anodized sample at 400 °C. It is noteworthy that the pseudohexagonal TT‐Nb_2_O_5_ is the most thermodynamically unstable crystal phase among several Nb_2_O_5_ phases,^43^ therefore, it is prone to be nitrided at elevated temperature. When increasing the nitridation temperature to 500 °C, the XRD pattern of the sample changes significantly. The extinction of Nb_2_O_5_ phase and the emergence of Nb_4_N_5_ phase (JCPDS No.51‐1327) demonstrate that nitridation of the as‐anodized sample starts to occur at 500 °C. Nb_4_N_5_ belongs to the tetragonal *I*4/*m* space group, and it is a defective NaCl‐type structure with niobium vacancies, which is closely related to δ‐NbN.^44^ With the further increase of nitridation temperature to 600 and 700 °C, the XRD peak positions remain nearly unchanged but the peaks intensity strengthens and peaks shape narrows, indicating better nitridation effect and crystallinity at higher temperature. XRD pattern of powder N‐700 sample (scratched from Nb substrate) clearly displays the pure Nb_4_N_5_ phase, with five diffraction peaks indexed as (211), (310)/(002), (420)/(312), (431), and (422) planes, respectively (Figure S1, Supporting Information). The reason why only Nb_4_N_5_ phase is found might be that the formation of Nb_4_N_5_ via the reaction of Nb_2_O_5_ and NH_3_ is thermodynamically and kinetically beneficial. The fabrication of Nb_4_N_5_ via different methods has also been reported in previous literatures.^44^, ^45^, ^46^

The field‐emission scanning electron microscopy (FE‐SEM) was adopted to characterize the morphologies of the Nb_4_N_5_ nanochanneled films (N‐700 sample). The top view SEM image in Figure 1c shows a highly nanoporous structure. As observed, the surface is rather coarse and the pores are not well organized, due to the nonuniform growth of the initial oxide layer in the anodization process, and the lattice shrinkage, which occurs during the structure transformation from niobium oxides to niobium nitrides. Generally, there are secondary sets of porous layers underneath the initial layer, which is composed of more organized nanosized pores. The oblique cross‐section view SEM image (Figure 1d) vividly exhibits the highly ordered nanochanneled structure of the Nb_4_N_5_ sample, with average channel diameter about 30–40 nm and thickness of side walls about 20–30 nm. The ordered Nb_4_N_5_ nanochannels can also be observed from the side view SEM image (Figure 1e). The entire Nb_4_N_5_ nanochanneled film possesses a thickness of about 17.4 μm.

The morphology and structure of the sample were further examined by transmission electron microscopy (TEM) (Figure S2, Supporting Information). The nanochannels appear ordered, straight but unsmooth feature, comprising many disintegrated particles. Well‐resolved lattice fringes of 0.216 nm are observed from the HR‐TEM image of Nb_4_N_5_ nanoparticles derived from the wall of Nb_4_N_5_ nanochannels, coincide with the (310) plane of tetragonal Nb_4_N_5_,^45^ demonstrating the presence of Nb_4_N_5_ phase. The ring structure of the selected area electron diffraction (SAED) pattern indicates the polycrystalline structure of the Nb_4_N_5_ nanochannels. The patterns of SAED are indexed as (211), (310)/(002), (420)/(312), and (431)/(422) planes of Nb_4_N_5_ on the basis of their *d*‐spacings, in accordance with the above‐mentioned XRD result, which further confirms the presence of Nb_4_N_5_ phase. Energy dispersive spectroscopy (EDS) data (Figure S3, Supporting Information) reveal that the sample has an Nb:N:O atomic ratio of 43.7:44.7:11.7. The relatively small amount of oxygen suggests the presence of some Nb_2_O_5_ on the Nb_4_N_5_ nanochannels surface.

**Figure 2:**
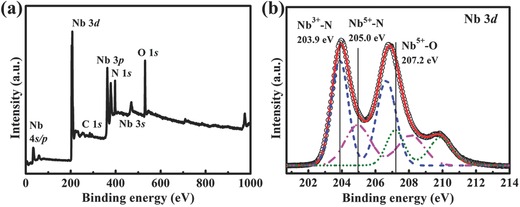
a) The survey XPS spectrum of Nb_4_N_5_ nanochannels. b) Nb 3d XPS spectrum. Black circles are the experimental data, which can be decomposed into a superposition of three fitting curves of Nb^3+^—N, Nb^5+^—N, and Nb^5+^—O. The red curve is the summation of the three decomposed curves.

X‐ray photoelectron spectroscopy (XPS) measurements were performed to study the surface chemical composition and oxidation state of the Nb_4_N_5_ nanochannels. XPS survey spectrum in **Figure** **2**a shows that Nb_4_N_5_ nanochannels are composed of four elements: Nb, N, O, and C, in accordance with the EDS result mentioned above. There is no other element found, indicting no impurity has been introduced into the sample during its preparation. The Nb 3d core level spectrum comprises two conspicuous doublets and an additional weak shoulder, as shown in Figure 2b. It is noteworthy that Nb_4_N_5_ comprises Nb^3+^ and Nb^5+^, thus the multiple peaks can be resolved as a sum of three different spin–orbit doublets. The best peak fit to the measured data involves the common contributions of Nb^3+^—N, Nb^5+^—N, and Nb_2_O_5_, which is consistent with the XRD and EDS results. The highest Nb 3d_5/2_ binding energy doublet at 207.2 eV can be assigned to Nb_2_O_5_, while the lowest binding energy doublet at 203.9 eV belongs to Nb^5+^—N in Nb_4_N_5_.^47^, ^48^, ^49^, ^50^, ^51^ Finally, the third component at 205 eV is identified as Nb^5+^—N. Their corresponding Nb 3d_3/2_ binding energies are 209.9, 206.6, and 208.1 eV, respectively. The fitting result reveals the presence of a small amount of niobium oxide on the surface of Nb_4_N_5_ nanochannels, in addition to the predominant Nb_4_N_5_, which is similar to that of TiN and VN nanowires.^15^, ^16^ The N 1s core level spectrum (Figure S4a, Supporting Information), which could be fitted with two peaks at around 397.2 and 399.2 eV, is assigned to Nb_4_N_5_ and a small amount of Nb—N—O,^47^, ^48^ respectively. Similarly, the O 1s core level spectrum (Figure S4b, Supporting Information) could also be fitted with two peaks located at around 530.7 and 532.3 eV. The former is characteristic of oxygen in Nb_2_O_5_, while the latter is associated with oxygen in Nb—N—O,^47^, ^52^ and even in some C—O and O—H bonds.

### 2.2 Electrochemical Properties Nb_4_N_5_ Nanochannels

**Figure 3:**
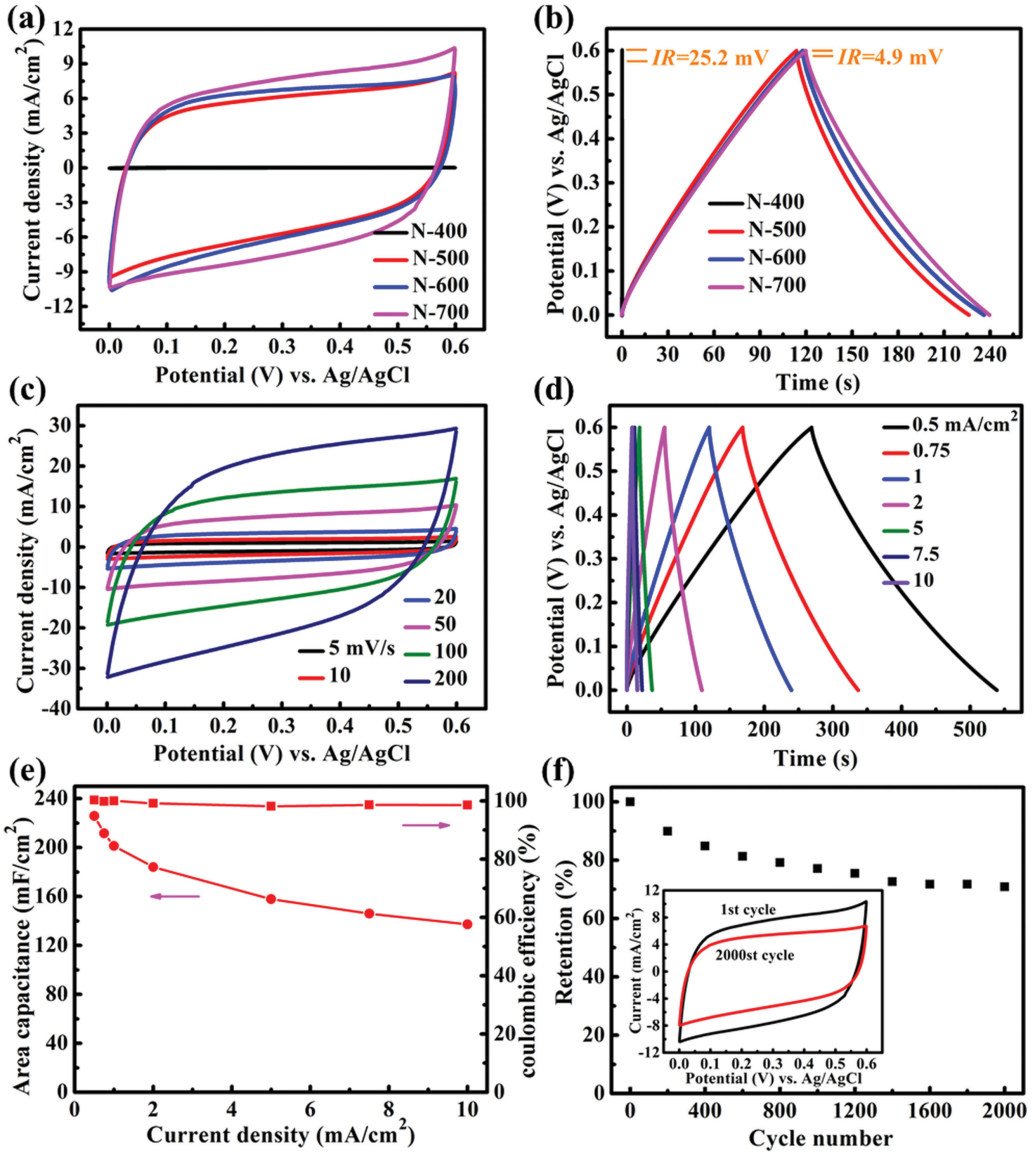
a) CV curves at a scan rate of 50 mV s^−1^ and b) GCD curves at a current density of 1 mA cm^−2^ of the samples nitrided at different temperatures. c) CV curves at different scan rates and d) GCD curves at different current densities of the N‐700 sample. e) Areal capacitance and coulombic efficiency as a function of current densities of N‐700 sample. f) Cycling performance of N‐700 sample at a scan rate of 50 mV s^−1^, inset shows the CV curves of the 1st and 2000th cycles.

To evaluate the electrochemical properties of samples nitride at different temperatures, a conventional three‐electrode system was utilized to conduct electrochemical measurements. The Nb_4_N_5_ (or Nb_2_O_5_) nanochannels attached on Nb foil substrates were used directly as the working electrode, with a Pt wire and an Ag/AgCl electrode as counter and reference electrodes, respectively, in 1 m H_2_SO_4_ aqueous solution. The cyclic voltammetry (CV) curves acquired from the samples in a potential range between 0 and 0.6 V (vs Ag/AgCl) at a scanning rate of 50 mV s^−1^ in 1 m H_2_SO_4_ are depicted in **Figure** **3**a. It is obvious that the reachable current densities of samples nitrided at 500–700 °C (Nb_4_N_5_ nanochannels) are much larger than that of sample nitrided at 400 °C (Nb_2_O_5_ nanochannels), demonstrating a significant increase in capacitance of Nb_4_N_5_ nanochannels. The poor capacitance of Nb_2_O_5_ nanochannels is confirmed by its CV curve with very low current density (Figure S5a, Supporting Information). The other three electrodes display approximately rectangular‐shaped and quite symmetrical CV curves as expected from an ideal capacitor. The N‐700 sample yields the highest current density, while the N‐500 and N‐600 samples display the comparable values.

Figure 3b compares the galvanostatic charge–discharge (GCD) curves of the four samples collected at a current density of 1 mA cm^−2^. The poor capacitance of N‐400 sample is observed from its transient GCD cycle (Figure S5b, Supporting Information). The enhanced capacitive behavior of N‐(500–700) samples is confirmed again by their substantially extended GCD cycles over N‐400 sample. The three potential–time curves with approximately equivalent charge time and discharge time are all nearly symmetric and a little nonlinear. We think that the capacitive response of Nb_4_N_5_ nanochannels can be attributed to both electrical double‐layer formation and pseudocapacitance arising from the Faradic reactions that occur on the surface of Nb_4_N_5_. The electrical double layer was formed by the hydrogen ions (protons) physisorbed on nonspecific sites, while Faradic reactions derive from the chemisorption of protons.

Chemisorption is likely to occur with the presence of the transition metal, which can undergo valence state transitions.^12^ Since Nb is a transition metal with variable oxidation states, and XPS results reveal the existence of Nb^5+^ in Nb_4_N_5_, it is favorable for the continuous chemisorption of protons throughout the potential range applied. No distinct peak related to the Faradaic reaction can be observed in the CV curves, probably because the charge/discharge process of active Nb_4_N_5_ is carried out at a pseudo‐constant rate over the entire potential range.^12^, ^53^ The mechanistic study of electrochemical reaction is to be discussed thereafter.

As depicted in Figure 3b, the *IR* drops of N‐400 and N‐700 samples are 25.2 and 4.9 mV at the current density of 1 mA cm^−2^, respectively. The equivalent series resistances (ESR) of N‐400 and N‐700 nanochannels electrodes are, respectively, calculated to be 12.6 and 2.45 Ω (ESR = *V*_drop_/2*I*, considering the 1 cm^2^ working area). Compared with Nb_2_O_5_ nanochannels, the apparently reduced ESR demonstrates the improved electrical conductivity of Nb_4_N_5_ nanochannels electrode. The symmetrical and linear *I*–*V* curve (Figure S6b, Supporting Information) of Nb_4_N_5_ nanochannels demonstrates the nearly metallic conductivity of Nb_4_N_5_.^23^ In contrast, the nonlinear and asymmetric *I–V* curve (Figure S6a, Supporting Information) of Nb_2_O_5_ nanochannels reflects the rectifying behavior of a Schottky junction, suggesting the semiconducting nature of Nb_2_O_5_ nanochannels.^23^ The fact that Nb_4_N_5_ nanochannels possess much higher electrical conductivity than Nb_2_O_5_ nanochannels is vividly depicted by the *I–V* curves. The conductivity of the Nb_4_N_5_ is measured to be 42.9 S cm^−1^ by four‐point probe method.

CV curves of the N‐700 Nb_4_N_5_ nanochannels electrode collected at different scan rates from 5 to 200 mV s^−1^ are shown in Figure 3c. Notably, the CV curves can still keep quasi‐rectangular shape as the scan rate goes up to 200 mV s^−1^, with only a small distortion from the ideal symmetrical rectangle shape, manifesting the excellent capacitive behavior and high rate capability. Figure 3d depicts the GCD curves of N‐700 Nb_4_N_5_ nanochannels at different current densities from 0.5 to 10 mA cm^−2^. The observation of nearly symmetric potential–time curves with quasi‐linear slopes at all current densities implies a high charge–discharge columbic efficiency and low polarization of the electrode. It is noteworthy that the nonlinearity of potential–time curves turns sharper with decreasing current density, which is the characteristic pseudo‐constant charge/discharge process over the entire potential window.^54^

Figure 3e presents the areal capacitances of N‐700 Nb_4_N_5_ nanochannels electrode calculated from its GCD curves. At the current density of 0.5 mA cm^−2^, the areal capacitance is 225.8 mF cm^−2^. When the current density increased 20‐fold to 10 mA cm^−2^, the areal capacitance drops to 137.2 mF cm^−2^, implying a desirable retention of 60.8% of the initial capacitance. From a practical point of view, the areal capacitance values are better than (or competitive with) those obtained from metal nitrides nanostructured film electrodes (even combining with other active materials), like mesoporous VN/CNT hybrid electrode (178 mF cm^−2^ at 1.1 mA cm^−2^),^55^ nanostructured TiN/CNT electrode (25.5 mF cm^−2^ at 100 mV s^−1^),^56^ coaxial polyaniline/TiN/polyaniline nanotube arrays (242 mF cm^−2^ at 0.2 mA cm^−2^),^23^ etc. The excellent areal capacitance and rate capability of the Nb_4_N_5_ nanochannels electrode are the concert of the favorable nanochanneled structure, the firm binder‐free connection between Nb_4_N_5_ and Nb substrate, the enhanced electrical conductivity, and the prominent electrochemical activity. In comparison, the N‐(400–600) samples, respectively, yield certain areal capacitances of 0.197, 224, and 231.3 mF cm^−2^ at 0.5 mA cm^−2^ (Figure S7, Supporting Information). Such a huge discrepancy between the areal capacitance of N‐400 sample and those of N‐(500–700) samples is attributed to the gap between the electrical conductivity of Nb_2_O_5_ and Nb_4_N_5_. Given the areal capacitances of 0.136, 113.9, and 122.7 mF cm^−2^ at 10 mA cm^−2^ current density for the N‐(400–600) samples, their retention are 68.9%, 50.8%, and 53%, respectively. Comparing the three N‐(500–700) samples, it can be derived that better crystallinity of Nb_4_N_5_ benefits the rate capability of Nb_4_N_5_ nanochannels electrode. Figure 3e exhibits the coulombic efficiency (CE), defined as the ratio of the total amount of discharge to charge calculated from the GCD curves. At all current densities, the Nb_4_N_5_ nanochannels electrode delivers a rather high CE around 99%, suggesting a good reversibility of the electrochemical reaction in the surface of Nb_4_N_5_ nanochannels.

In order to examine the cycling stability of Nb_4_N_5_ nanochannels, CV measurement was conducted on N‐700 sample for successive 2000 cycles at the scan rate of 50 mV s^−1^. The capacitance retention and the CV curves of the first and 2000th cycles, are present in Figure 3f. The performance of the Nb_4_N_5_ electrode undergoes a relatively distinct decay in the first 1600 cycles, and then tends to be steady. After 2000 cycles, 70.9% of the initial capacitance remains, which is not outstanding but better than that of most metal nitrides ever reported, such as TiN, VN, and Mo_2_N.^11^, ^12^, ^17^

Similar with other metal nitrides, the non‐negligible capacitance loss of Nb_4_N_5_ nanochannels derives from an irreversible conversion from Nb_4_N_5_ to nonactive Nb_2_O_5_ by electrochemical oxidation in the presence of water and oxygen. The capacitance loss can also be observed from the two CV curves of the first and last cycles, which have the analogous shape more or less. However, the latter possesses an obviously smaller reachable current. XRD pattern measured after cycling test delivers apparently lower peaks of Nb_4_N_5_ phase than that before cycling (Figure S8, Supporting Information), demonstrating the depletion of Nb_4_N_5_ caused by electrochemical oxidation in the long‐term test. The variation in Nb 3d core level XPS measured before and after cycling clearly exhibits the surface oxidation condition of Nb_4_N_5_ nanochannels (Figure S9, Supporting Information). The reduced peak around 203.9 eV (Nb 3d_5/2_ in Nb_4_N_5_) and enhanced peak around 209.9 eV (Nb 3d_3/2_ in Nb_2_O_5_) of the XPS after cycling compared with that before cycling, unambiguously indicates the considerable oxidation of Nb_4_N_5_ into Nb_2_O_5_.The capacitance retentions of N‐(400–600) samples are calculated to be 122.9% (attributed to self‐activation process), 62.3%, and 70.4%, respectively (Figure S10, Supporting Information). Thus, higher nitridation temperature produces better cycling stability for Nb_4_N_5_ nanochannels, which is attributed to the better crystallinity. Apparently, the cycling stability of our Nb_4_N_5_ nanochannels is insufficient to support their practical applications in high‐performance supercapacitors. Thus, effective optimization processes are required to be introduced to improve their cycling stability, exactly as our follow‐up work described later.

**Figure 4:**
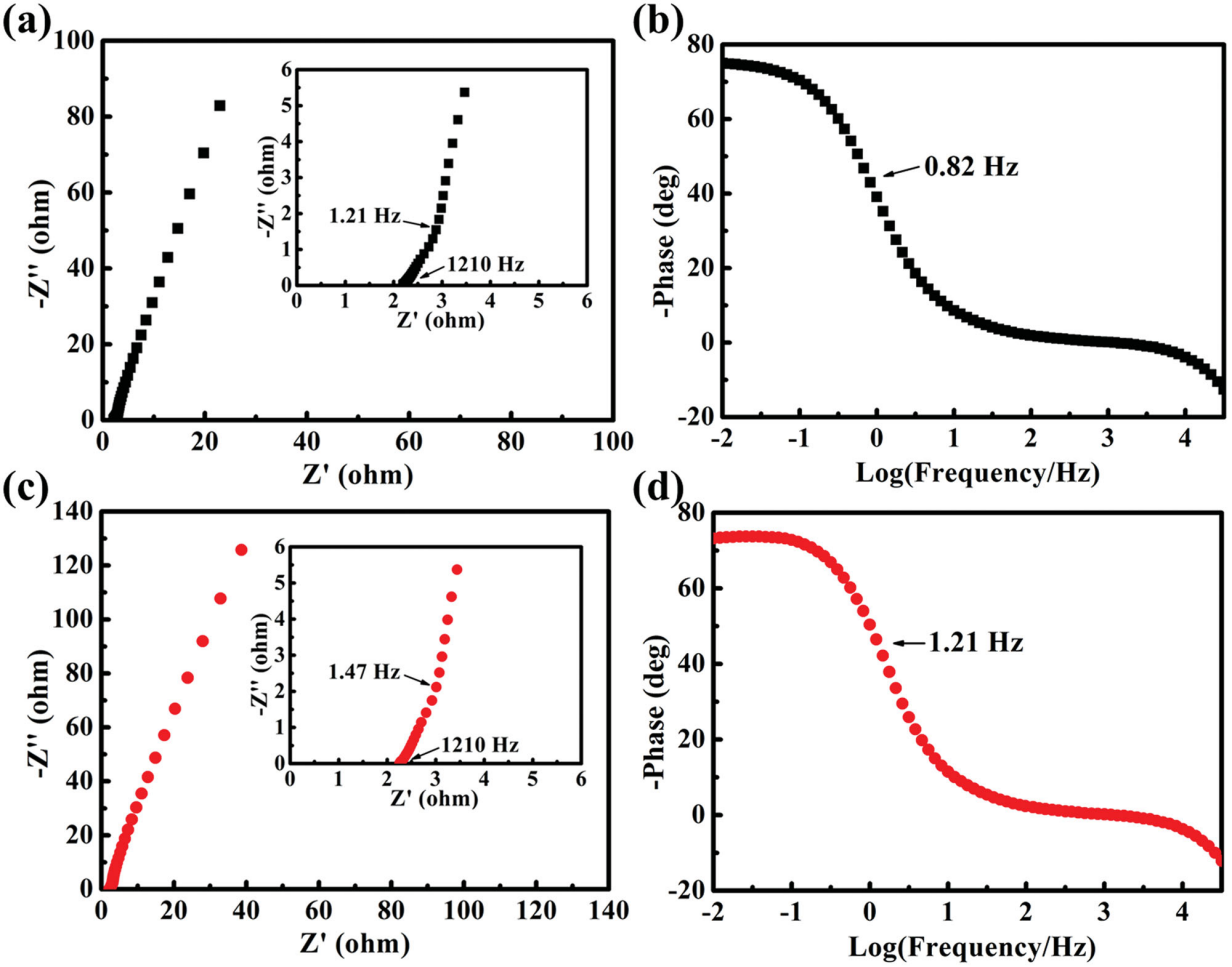
a) Nyquist plots and b) Bode phase angle plots of Nb_4_N_5_ nanochannels electrode before cycling. c) Nyquist plots and d) Bode phase angle plots after 2000 cycles. Insets are zoom‐in of the high frequency regions.

In order to further investigate the electrochemical behavior of Nb_4_N_5_ nanochannels, electrochemical impedance spectra (EIS) measurements were performed at open circuit potential with an amplitude of 5 mV before and after cycling. Typical Nyquist plots with frequencies ranging from 0.01 to 100 KHz are shown in **Figure** **4**a. The bulk solution resistance (*R*_s_) is estimated to be about 2.2 Ω. The absence of an impedance arc in the high‐frequency region indicates very low charge transfer resistances (*R*_ct_) of Nb_4_N_5_ nanochannels, due to the high solvated ion accessible surface area of their nanochanneled structure and the intrinsic outstanding electrical conductivity and electrochemical activity of Nb_4_N_5_ material. At low frequencies, the sample exhibits typical capacitive behavior, where an almost vertical line is observed on the Nyquist plot and a phase angle close to −80° on the Bode plot (Figure 4b). The knee frequency is about 1.21 Hz, showing that a pure capacitive behavior can be obtained and most stored energy in Nb_4_N_5_ nanochannels is accessible at frequencies below this frequency.^57^ The characteristic frequency *f*_0_ for a phase angle of ‐45° is about 0.82 Hz for the electrode. This frequency marks the point, at which the resistive and capacitive impedances are equal to each other. The corresponding time constant *τ*_0_ (1/*f*_0_) equals 1.22 s, which is the minimum time needed to discharge all the energy with an efficiency of more than 50%, is much shorter than 10 s for the conventional activated carbon supercapacitors.^58^ Both the Nyquist plot and Bode plot measured after 2000 CV cycles (Figure 4c,d) resemble those prior to cycling, without distinct change on both plots. The Nyquist and Bode plots of the N‐400 sample (Figure S11, Supporting Information) depict the large charge transfer resistance and poor capacitive behavior of Nb_2_O_5_ nanochannels.

### 2.3 Mechanistic Studies on the Capacitive Behavior of Nb_4_N_5_ Nanochannels

In order to explore the capacitive mechanism of Nb_4_N_5_ nanochannels, 1 m H_2_SO_4_ and 1 m Na_2_SO_4_ were chosen as electrolytes to conduct electrochemical test, respectively. Although the two CV curves at 50 mV s^−1^ present similar quasi‐rectangular shapes (Figure S12a, Supporting Information), the capacitive current in H_2_SO_4_ is remarkably larger than that in Na_2_SO_4_. Similarly, the discharge time of Nb_4_N_5_ nanochannels electrode is much longer in H_2_SO_4_ electrolyte than that in Na_2_SO_4_ electrolyte at the same current density (Figure S12b, Supporting Information). At 0.5 mA cm^−2^, the areal capacitance of Nb_4_N_5_ nanochannels electrode in Na_2_SO_4_ is calculated to be 138.7 mF cm^−2^, which is 61.4% of that in H_2_SO_4_ (225.8 mF cm^−2^). Considering the huge difference of H^+^ ion concentration in these two electrolytes, the large amount of H^+^ is responsible for the higher areal capacitance of Nb_4_N_5_ nanochannels in H_2_SO_4_. The pseudocapacitance can be enhanced by the chemisorbed protons. Hence, we think that the pseudocapacitance of Nb_4_N_5_ is originated from the intercalation/chemisorption of the proton into the solid phase. In order to evaluate the actual contribution of EDLC and pseudocapacitance, Nb_4_N_5_ nanochannels two‐electrode system measurements were performed both in 1 m H_2_SO_4_ and 1 m LiClO_4_/acetonitrile anhydrous electrolyte. As shown by the CV curves (50 mV s^−1^) and GCD curves (1 mA cm^−2^) (Figure S13, Supporting Information), the capacitive performance in H_2_SO_4_ is much better than in anhydrous electrolyte. At 1 mA cm^−2^, the areal capacitance of the two‐electrode system is calculated to be 95.1 mF cm^−2^ in H_2_SO_4_, and 47.2 mF cm^−2^ in LiClO_4_/ACN electrolyte. Assuming the capacitance in anhydrous electrolyte all derived from EDL, the contribution of EDLC is 49.6% and pseudocapacitance contributes the other part.

**Figure 5:**
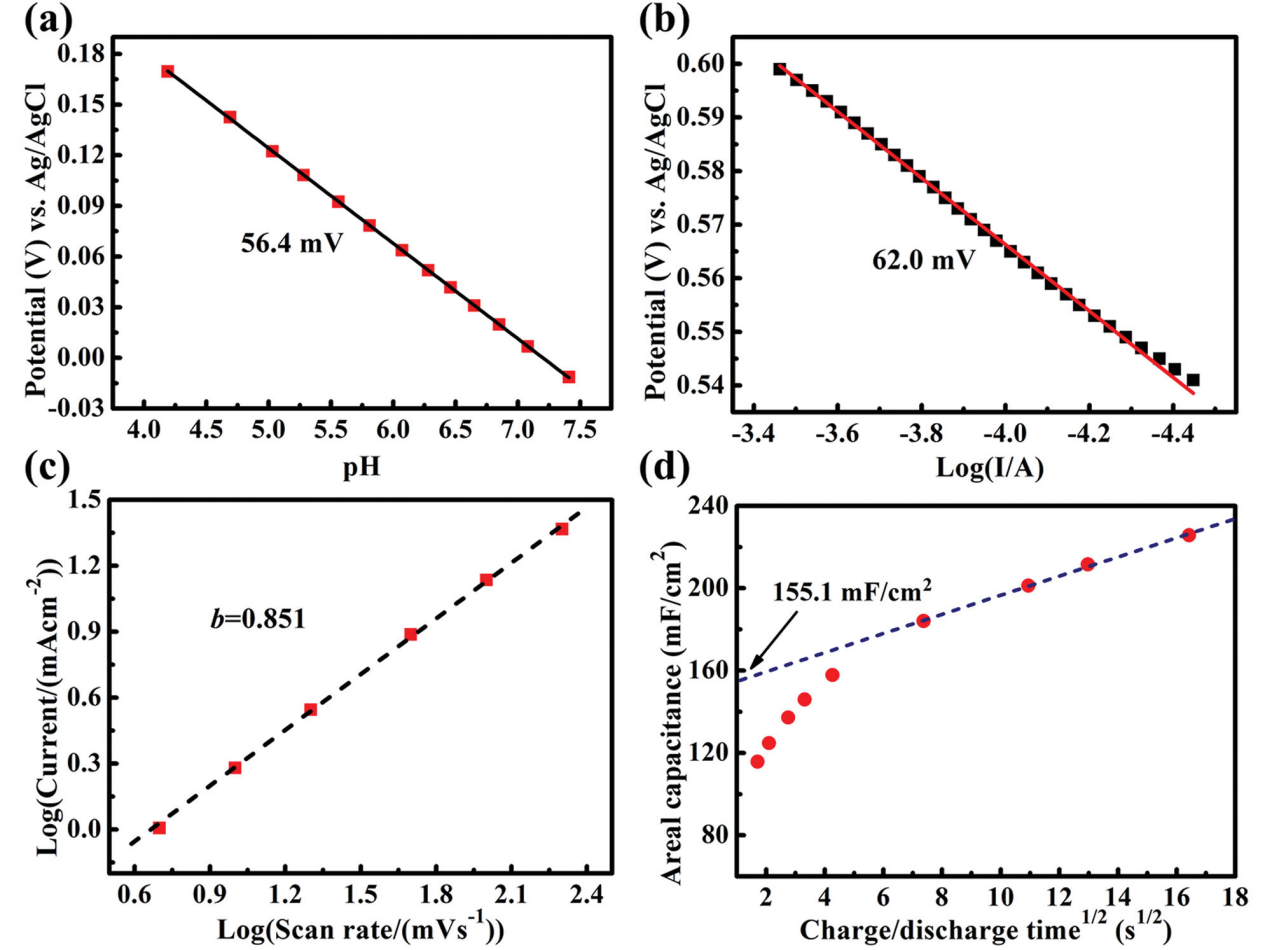
a) pH dependence of steady‐state Nb_4_N_5_ nanochannels electrode potential at constant current density (*i* = 0.5 μA cm^−2^) in 1 m KH_2_PO_4_ solution using 1 m KOH to raise the pH. b) Electrode potential against current density at Tafel zone measured in 1 m H_2_SO_4_. c) *b*‐value determination of the currents of anodic CV curves (at 0.3 V_Ag/AgCl_ in the CV sweeps from 5 to 200 mV s^−1^ in 1 m H_2_SO_4_). d) The capacitance versus (discharge time)^1/2^.

We postulate reversible proton incorporation/chemisorption into the Nb_4_N_5_ nanochannels electrode to cause pseudocapacitance with the valence decrease of Nb ions. Thus, an electrode potential Φ at a proton activity *a*(H*^+^*) providing a driving force Δ*G* = –zFϕ +*RT*ln(*a*(H^+^)) for a proton‐incorporation/chemisorption reaction which involves *z* electrons, the steady‐state reaction current *i* ∝ exp(−Δ*G*/*RT*) is as follows^59^

(1) $$i=i_{o}a{\left(H^{+}\right)}^{-1}\exp\left(\frac{zF\phi }{RT}\right)$$

where *F* is the Faraday constant, *i*_o_ is a proportionality constant, and *RT* has its usual meaning. Comparing the slopes in the Tafel plots (**Figure** **5**a,b) with the theoretical value (2.3*RT*/*F* = 59.2 mV decade^–1^), we determine *z* = 1 for Nb_4_N_5_. This can be explained by that in Nb_4_N_5_ comprising copious +5 valence Nb ions, each reactive site receives one proton and one electron converting it to NH. The mechanism of the proton incorporation is supported by the fact that the capacitance is higher in an acidic aqueous electrolyte than that in a neutral aqueous electrolyte and in an anhydrous electrolyte.

The kinetic information obtained from CV curves should obey the power law with the relationship^60^

(2) $$i=av^{b}$$

where *i* is the peak current (mA), *v* is the sweep rate (mV s^−1^), *a* and *b* are coefficients. The calculated *b*‐value can be used to distinguish the charge storage whether it arises from capacitive or diffusion‐controlled processes. While *b* = 0.5 represents a semi‐infinite diffusion‐controlled process, *b* = 1 symbolizes capacitive behavior. By fitting a log(anodic current)–log(scan rate) plot (Figure 5c), a *b*‐value of 0.851 can be obtained, which indicates that a surface‐controlled capacitive electrode process dominates the Nb_4_N_5_ nanochannels electrode.^31^

According to the theory by Trasatti and co‐workers,^61^ the capacity can be characterized by the dependence of capacitance (*C*) on the scan rates (*v*)

(3) $$C=C_{v=\infty }+\mathrm{constant}(v^{-1/2})$$

where *C_v_*_=__∞_ represents capacitive (outer surface) contribution, constant(*v* ^−1/2^) represents diffusion‐controlled capacity. In the plot of capacity versus *v* ^−1/2^ (replaced by (discharge time)^1/2^, Figure 5d), the extrapolation of the linear fit to the data to the *y*‐intercept (time = 0) gives *C_v_*_=__∞_ (155.1 mF cm^−2^), as the contribution of the so‐called outer surface. At 0.5 mA cm^−2^, the outer surface contributes ≈64% of the total capacity. The rest is due to the intercalation of protons into the subsurface of Nb_4_N_5_ nanochannels.

### 2.4 Improvement of the Cycling Stability of Nb_4_N_5_ Nanochannels with a Thin Carbon Coating

Due to the irreversible electrochemical oxidation of Nb_4_N_5_ during the charging/discharging process, the cycling stability of our Nb_4_N_5_ nanochannels is not satisfactory, with 29.1% capacitance loss after 2000 CV cycles. Previous studies showed that an ultrathin and stable amorphous carbon protective coating can effectively stabilize the electrochemical performance of metal nitride (TiN and VN) and polymer (polyaniline and polypyrrole) electrodes, using glucose as the carbon source.^20^, ^62^ Dopamine is also proved to be a favorable agent for carbon coating to ameliorate the performance of electrode materials of supercapacitor and Li‐ions battery, such as MnO_2_ and Fe_3_O_4_.^38^, ^40^

**Figure 6:**
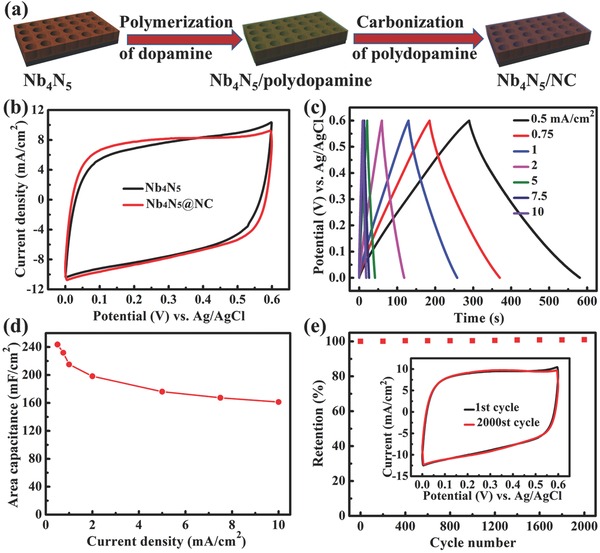
a) Schematic diagram illustrating the fabrication of N‐doped carbon coating on the surface of Nb_4_N_5_ nanochannels. b) CV curves of Nb_4_N_5_ nanochannels and Nb_4_N_5_@NC nanochannels at a scan rate of 50 mV s^−1^. c) GCD curves at different current densities and d) areal capacitance as a function of current densities of Nb_4_N_5_@NC nanochannels. e) Cycling performance of Nb_4_N_5_@NC nanochannels electrode at 50 mV s^−1^, inset shows the CV curves of the 1st and 2000th cycles.

Based on the positive results of aforementioned studies, we intend to improve the cycling stability of our Nb_4_N_5_ nanochannels through the introduction of carbon coating to the surface, employing dopamine as the carbon source. The fabrication process of N‐doped carbon coated Nb_4_N_5_ nanochannels (abbreviated as Nb_4_N_5_@NC nanochannels) is schematically illustrated in **Figure** **6**a. For a typical procedure by reference to previous report with some modification,^40^ obtained Nb_4_N_5_ nanochannels were immersed in an aqueous solution (pH ≈ 8.5). Subsequently, the dopamine monomer translates into polydopamine through an oxidization and cyclization reaction after overnight standing. Afterward, the core–shell structured Nb_4_N_5_@polydopamine nanochannels are transformed into Nb_4_N_5_@NC nanochannels by thermal annealing in Ar at 500 °C. The details about the experimental procedure and general coating mechanism are clearly described in the Experimental Section.

The TEM images of Nb_4_N_5_@NC composites (Figure S14, Supporting Information) derived from ultrasonically smashed Nb_4_N_5_@NC nanochannels, show that Nb_4_N_5_ nanoparticles are coated with a thin amorphous carbon layer with different thickness (1–2 nm), displaying a typical core–shell nanostructure. It is noted that no extra phase is introduced into the sample or the Nb_4_N_5_ phase deteriorates during the carbon‐coating process, according to the XRD pattern of Nb_4_N_5_@NC nanochannels in comparison with that of Nb_4_N_5_ specimen (Figure S15, Supporting Information). Figure 6b compares the CV curves at 50 mV s^−1^ of Nb_4_N_5_ and Nb_4_N_5_@NC electrodes. Though no apparent change occurs in the reachable current, the shape of CV curve of Nb_4_N_5_@NC specimen becomes a little more rectangular, indicating the enhanced electrical conductivity and capacitive behavior with the additive carbon coating on the surface. The GCD curves in Figure 6c are almost completely symmetric, indicating a coulombic efficiency very close to 100% at all current densities. The areal capacitance at 0.5 mA cm^−2^ is calculated to be 243.6 mF cm^−2^, which is a little higher than that (225.8 mF cm^−2^) of Nb_4_N_5_ without carbon coating. Then, it drops to 161.3 mF cm^−2^ with the increase of current density to 10 mA cm^−2^, delivering a good rate capability with a retention of 66.2%, higher than that (60.8%) of Nb_4_N_5_. The rate capability is associated with the ion diffusion rate in the electrode and the conductivity of electrode. Considering the similar nanochanneled structure of the Nb_4_N_5_@NC nanochannels and Nb_4_N_5_ nanochannels, they should possess similar ion diffusion rate. Therefore, the enhanced electrical conductivity due to the conductive carbon coating should account for the improved rate capability of Nb_4_N_5_@NC nanochannels. The series resistance (1.6 Ω) calculated from the *IR* drop (3.2 mV) at 1 mA cm^−2^ current density (Figure S16, Supporting Information) is also lower than that of Nb_4_N_5_ nanochannels (2.4 Ω) mentioned above, confirming the increased electrical conductivity of Nb_4_N_5_@NC nanochannels again.

To evaluate the influence of the carbon coating on the cycling stability of Nb_4_N_5_@NC nanochannels, CV measurement was conducted for successive 2000 cycles at the scan rate of 50 mV s^−1^, exactly like the test method for Nb_4_N_5_ nanochannels. As shown in Figure 6e, the Nb_4_N_5_@NC nanochannels electrode exhibits prominent cycling stability, holding a perfect retention of nearly 100% at every cycle number. Contrary to the common capacitance loss, the capacitance of Nb_4_N_5_@NC nanochannels increases gradually in the long cycle process. To be more precise, the total increase rate after 2000 cycles is 0.93%, which should be attributed to self‐activation process. The two CV curves before and after cycling overlap almost completely, reconfirming the exceedingly excellent cycling stability of Nb_4_N_5_@NC nanochannels again. Compared with the inferior stability of Nb_4_N_5_ nanochannels (29.1% loss after 2000 cycles), the remarkable stability amelioration of Nb_4_N_5_@NC sample is contributed from the ultrathin carbon coating on the surface, which can effectively protect the inner Nb_4_N_5_ active material from undesired reactions with H_2_O and O_2_, thus dramatically suppressing the electrochemical oxidation of Nb_4_N_5_. The improvement of cycling stability of Nb_4_N_5_@NC nanochannels can also be demonstrated by Nb 3d core level XPS measured before and after cycling (Figure S17, Supporting Information). There is no obvious change occurring in the peak intensity around 203.9 eV (Nb 3d_5/2_ in Nb_4_N_5_) and that around 209.9 eV (Nb 3d_3/2_ in Nb_2_O_5_) of the XPS after cycling compared with that before cycling, confirming the outstanding stability of active Nb_4_N_5_ protected by the carbon coating. The C 1s XPS spectra before and after cycling test (Figure S18, Supporting Information) are essentially the same, which is evidence of the electrochemical stability of the carbon coating. Moreover, there is almost no obvious change occurring in the Nyquist plot Nb_4_N_5_@NC nanochannels (Figure S19, Supporting Information) after cycling, manifesting the retentive electrical conductivity and capacitive behavior of Nb_4_N_5_@NC nanochannels.

### 2.5 Electrochemical Performance of Nb_4_N_5_@NC Nanochannels Two‐Electrode Cell

**Figure 7:**
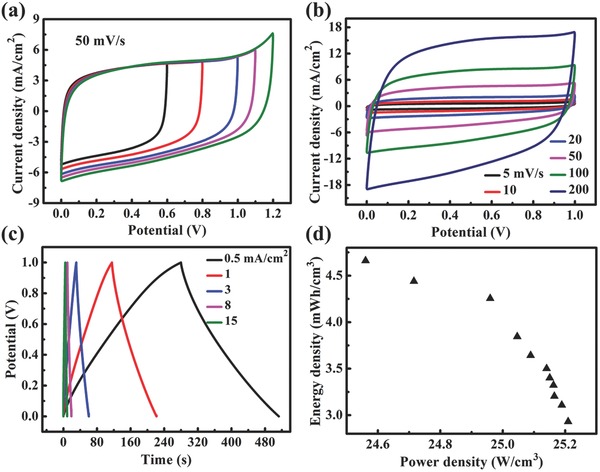
a) CV curves of Nb_4_N_5_ nanochannels two‐electrode cell within different potential window. b) CV curves of Nb_4_N_5_ nanochannels two‐electrode cell at different scan rates within 0–1 V. c) GCD curves at different current densities. d) Ragone plot (energy density vs power density) for the device at different charge/discharge current densities.

To further investigate the possibility for practical applications of the Nb_4_N_5_@NC nanochannels, electrochemical performance of Nb_4_N_5_@NC nanochannels two‐electrode cell was measured. To confirm the favorable operating potential, the CV curves at 50 mV s^−1^ were measured with different potential windows, as shown in **Figure** **7**a. The aqueous Nb_4_N_5_@NC nanochannels symmetric supercapacitors demonstrate an ideal capacitive behavior without obvious polarization curves, even at the potential window as large as 1.1 V. The enlargement of the cell voltage will be a critical factor to improve the energy density of the Nb_4_N_5_@NC nanochannels supercapacitors.

Figure 7b shows the CV curves of Nb_4_N_5_@NC nanochannels electrodes in a symmetrical two‐electrode configuration within the potential range between 0 and +1 V. The approximate rectangle shape of the symmetric cell is indicative of an ideal capacitive behavior. GCD measurements are critical in the analysis and prediction of the active materials' performance under practical operating conditions. It can be seen that the GCD curves in Figure 7c are nearly linear and symmetrical, which is another typical characteristic of ideal capacitor behavior. The ESR value of the cell is also determined from the linear fit relation between *IR* voltage drop and discharge current density (*IR* drop = 7.5 × 10^−5^ + 0.0057*I*) (Figure S20, Supporting Information), which is rather small and favors high discharge power delivery in practical applications. The specific capacitance of the cell at the current density of 0.5 mA cm^−2^ is calculated to be 116.45 mF cm^−2^, corresponding to 232.9 of a single electrode when multiplied by a coefficient of 2, which is slightly smaller than the measured value from the three‐electrode system. Energy density and power density have been widely used to evaluate the performance of SCs. The Ragone plot in Figure 7d shows the volumetric energy density and power density of the Nb_4_N_5_@NC nanochannels device. According to the Ragone plot, the highest energy density is 4.66 mWh cm^−3^ with a power density of 24.56 W cm^−3^, and remains 2.93 mWh cm^−3^ at 25.21 W cm^−3^.

## 3 Conclusions

We have prepared highly ordered Nb_4_N_5_ nanochannels by anodizing of Nb foil, followed by subsequent annealing in ammonia atmosphere, and demonstrated their electrochemical performance as supercapacitor electrodes for the first time. An areal capacitance of 225.8 mF cm^−2^ at a current density of 0.5 mA cm^−2^ is achieved in 1 m H_2_SO_4_ electrolyte, which is among the best level of metal nitrides nanostructured film electrodes. The favorable structure of Nb_4_N_5_ nanochannels, the outstanding electrical conductivity, and the prominent electrochemical activity are collectively responsible for the excellent capacitive behavior. Faradaic pseudocapacitance is confirmed by the mechanistic studies, deriving from the proton incorporation/chemisorption reaction owing to the copious +5 valence Nb ions in Nb_4_N_5_. After 2000 CV cycles, 70.9% of initial capacitance remains. Furthermore, by coating a carbon layer, excellent cycling stability is achieved from the Nb_4_N_5_@NC nanochannels electrode, with nearly 100% capacitance retention after 2000 CV cycles. In perspective, the reported Nb_4_N_5_ nanochannel structure in this work can also serve as supports for deposition of other active capacitive materials in order to construct hybrid electrodes, and potential applications for other energy‐related applications.

## 4 Experimental Section

*Materials*: Nb foils (99.7%, 127 μm in thickness) were purchased from Alfa Aesar. K_2_HPO_4_ and glycerol were purchased from Sinopharm Chemical Reagent Co. Ltd, China. 2‐amino‐2‐hydroxymethyl‐propane‐1,3‐diol (Tris, 99%) and dopamine hydrochloride (98%) were purchased from Aladdin Industrial Inc., China.

*Preparation of Nanochannels*: Niobium foils with dimension of 2 × 1 cm were cleaned by sonication in soap solution, acetone, ethanol, and deionized water successively. Then the foils were chemically polished in a mixture of HF/HNO_3_/H_2_O (1:4:5) for 10 min to obtain smooth surface. Finally, the samples were dried by nitrogen gas.

The anodization was conducted in the electrolyte containing 10 wt% K_2_HPO_4_ in anhydrous glycerol. Prior to anodization, the electrolyte was held at 180 °C for 4 h to reduce the water content. The target area for anodization was set as 1 × 1 cm on single side of the Nb foil, and the undesired part was protected by insulating tape. The samples were anodized at 20 V supplied by a DC power supply (INTERLOCK IPD‐20001SLU, China) for 15 min, at 180 °C as suggested previously, to produce ≈17.4 μm thick Nb_2_O_5_ nanochannels. Anodization was carried out in a two‐electrode system configuration with a Pt plate as the cathode electrode.

The as‐anodized samples were rinsed with deionized water and ethanol, and spontaneously dried in the air for several hours. Then they were nitrided in NH_3_ at a temperature range of between 400 and 700 °C for 4 h (ramp up/down of 10 °C min^–1^). After cooling down to the room temperature spontaneously, the samples were taken out and Nb_4_N_5_ nanochannels were obtained.

*Preparation of N‐Doped Carbon Coated Nb_4_N_5_ Nanochannels*: The preparation of N‐doped carbon coating on the surface of Nb_4_N_5_ nanochannels was according to the literature with some modification. For a typical procedure, 30 mg Tris was dissolved in 24 mL deionized water to form Tris buffer, and the pH was accurately adjusted to about 8.5 with pH meter. Subsequently, 24 mg dopamine hydrochloride was added to Tris buffer under stirring. Afterward, the sample of Nb_4_N_5_ nanochannels attached on Nb substrate was directly immersed into the dopamine aqueous solution, and allowed to stand statically for 12 h at room temperature. Then the sample was removed from the solution and washed with deionized water, and then dried at 50 °C in an electric oven for 12 h. The obtained sample was heated to 150 °C in Ar atmosphere and kept at this temperature for 1 h, and then further heated to 500 °C with kept for 4 h.

*Material Characterization*: To investigate the microstructure and composition of the samples, FE‐SEM (Hitachi S‐4800), TEM (JEOL JEM‐2100F), XRD (Bruker D8 Advance), and XPS (PHI 5000C ESCA System) with MgKα X‐ray (*hν* = 1253.6 eV) at 14 kV were employed. To measure the electrical conductivity of Nb_4_N_5_ by four‐point probe method (ACCENT HL‐5500 Hall effect testing instrument), the Nb_4_N_5_ powder was scratched from the substrate, cold‐pressed into a disk sample, and annealed in NH_3_ at 700 °C for 3 h.

To characterize the electrochemical performance of the samples, a conventional three‐electrode system was utilized to conduct electrochemical measurements. The Nb_4_N_5_ nanochannels attached on Ti substrates were used directly as the working electrode, with a Pt wire and an Ag/AgCl (KCl saturated) electrode as counter and reference electrodes, respectively, in 1 m H_2_SO_4_ aqueous solution. EIS, CV, GCD, and Tafel tests were carried out by an electrochemical workstation (CHI660B, CH Instruments). The cycling stability was tested by CV measurements at a constant scan rate of 50 mV s^−1^ for 2000 cycles.

## Supporting information

As a service to our authors and readers, this journal provides supporting information supplied by the authors. Such materials are peer reviewed and may be re‐organized for online delivery, but are not copy‐edited or typeset. Technical support issues arising from supporting information (other than missing files) should be addressed to the authors.

Supplementary
