# Supplementary material for: Niobium Nitride Nb4N5 as a New High‐Performance Electrode Material for Supercapacitors
Source: Adv Sci (Weinh). 2015 Jul 15;2(12):1500126. doi: 10.1002/advs.201500126 (PMC5115299; doi:10.1002/advs.201500126)
Supplement: Supplementary file 1 — Supplementary [file ADVS-2-0i-s001.pdf]

Copyright WILEY-VCH Verlag GmbH & Co. KGaA, 69469 Weinheim, Germany, 2015.

ADVANCED SCIENCE
Open Access

Supporting Information

for *Adv. Sci.*, DOI: 10.1002/advs.201500126

Niobium Nitride Nb_4N_5 as a New High-Performance Electrode Material for Supercapacitors

*Houlei Cui, Guilian Zhu, Xiangye Liu, Fengxin Liu, Yian Xie, Chongyin Yang, Tianquan Lin, Hui Gu, and Fuqiang Huang**

Supporting Information

Niobium Nitride Nb_4N_5 as a New High-performance Electrode Material for Supercapacitors

*Houlei Cui, Guilian Zhu, Xiangye Liu, Fengxin Liu, Yian Xie, Chongyin Yang, Tianquan Lin, Hui Gu and Fuqiang Huang**

Calculations

For Single Electrode

The areal capacitance was calculated from GCD curves by the following equation:

$$C_a = I\Delta t/(S\times\Delta U)$$

where C_a (mF cm^{-2}) is the areal capacitance of the electrode, I (mA) is the discharge current, Δt (s) is the discharge time, ΔU (V) is the potential window (0.6 V) during the discharge process, S (cm^2) is the surface area of working electrode.

The areal capacitance can also be calculated from CV curves according to the following equation:

$$C_a = (I\mathrm{d}V)/(2v\times\Delta U\times S)$$

where $\int I\mathrm{d}V$ is the integral areal of one CV cycle, v is the potential scan rate, ΔU is the potential window, S is the surface area of working electrode.

For two-electrode symmetrical device

The capacitance of the device (C) was estimated from the slope of the discharge curve using the following equations:

$$C = I\Delta t/\Delta U$$

where C (mF) is the capacitance of the device , I (mA) is the discharge current, Δt (s) is the discharge time, ΔU (V) is the potential window (1 V) during the discharge process.

Volumetric energy density (E, mWh cm^{-3}), equivalent series resistance (ESR, ohm) and power density (P, mW cm^{-3}) of the device were obtained from the following equations:

$$E = 0.5C\Delta U^2/3600V = 0.5C\Delta U^2/(3600\times S\times T)$$

$$ESR = IR_{\mathrm{drop}}/2I$$

$$P = \Delta U^2/(4ESR\times V) = \Delta U^2/(4ESR\times S\times T)$$

where E (mWh cm^{-3}) is the volumetric energy density, C (mF) is the device capacitance calculated from GCD curve, ΔU (1V) is the voltage window, V (cm^3) is the volume of active material, S (cm^2) is the surface area of the device, T (0.00174cm×2) is the thickness of Nb_4N_5 active material on both electrodes. ESR (ohm) is the internal resistance of the device. P (mW cm^{-3}) is the volumetric power density.

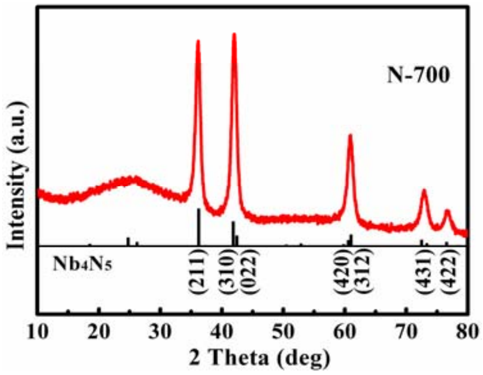

Figure S1. XRD pattern of powder N-700 sample (scraped off from Nb substrate).

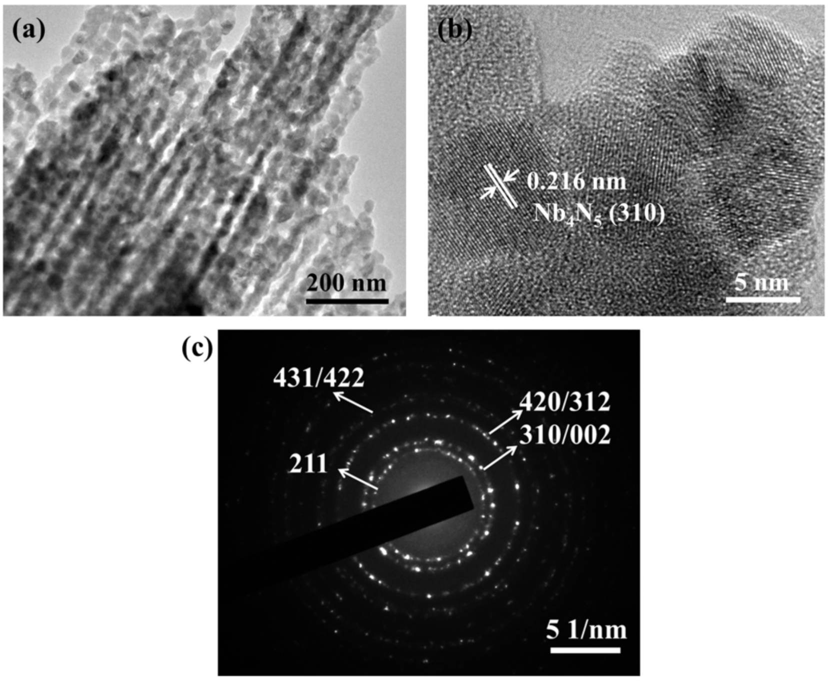

Figure S2. (a) TEM and (b) HR-TEM and (c) selected area electron diffraction (SAED) pattern of the Nb_4N_5 nanochannels.

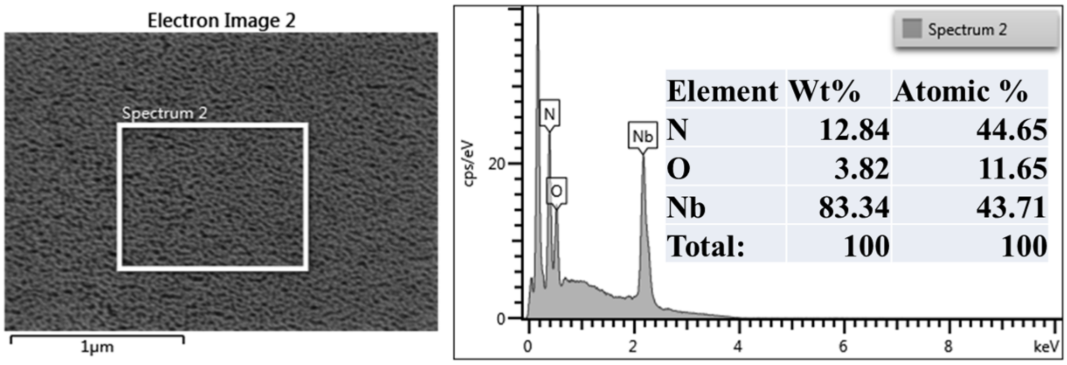

Element	Wt%	Atomic %
N	12.84	44.65
O	3.82	11.65
Nb	83.34	43.71
Total:	100	100

Figure S3. EDS scanned spectrum collected from the surface of Nb_4N_5 nanochannels.

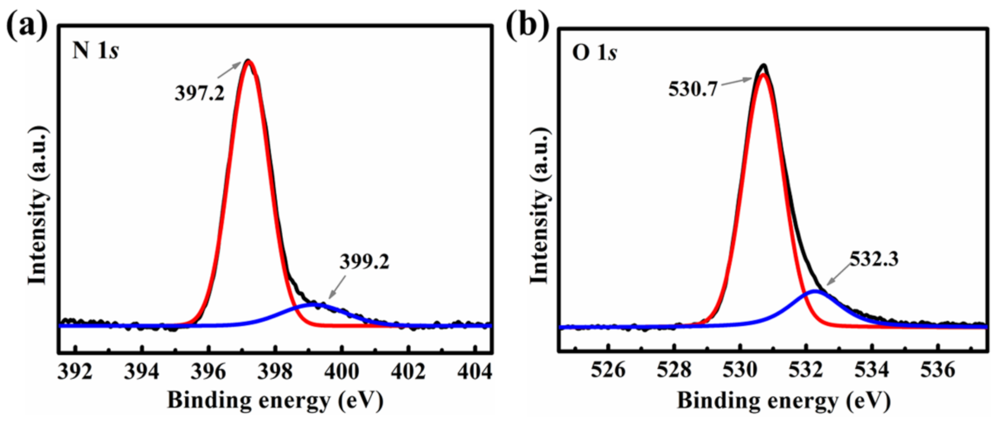

Figure S4. (a) N 1s and (b) O 1s XPS spectra in Nb_4N_5 nanochannels.

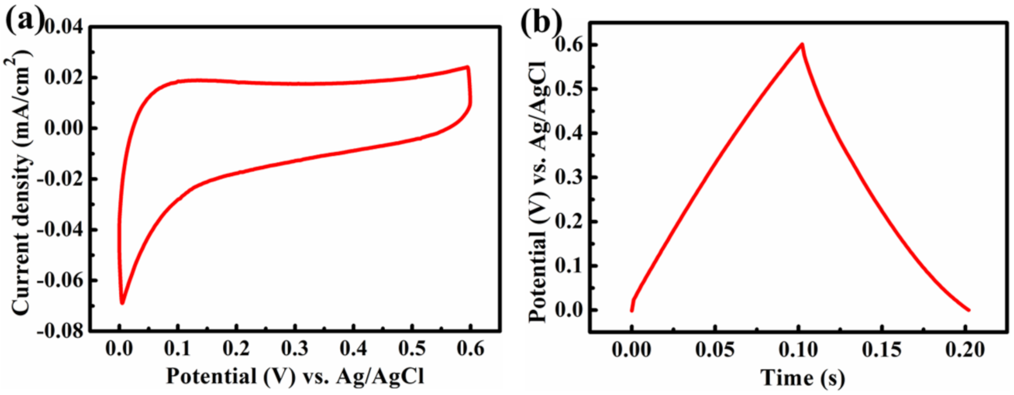

Figure S5. (a) CV curve at a scan rate of 50 mV s^{-1} and (b) GCD curve at a current density of 1 mA cm^{-2} of the N-400 sample (Nb_2O_5) for enlargement.

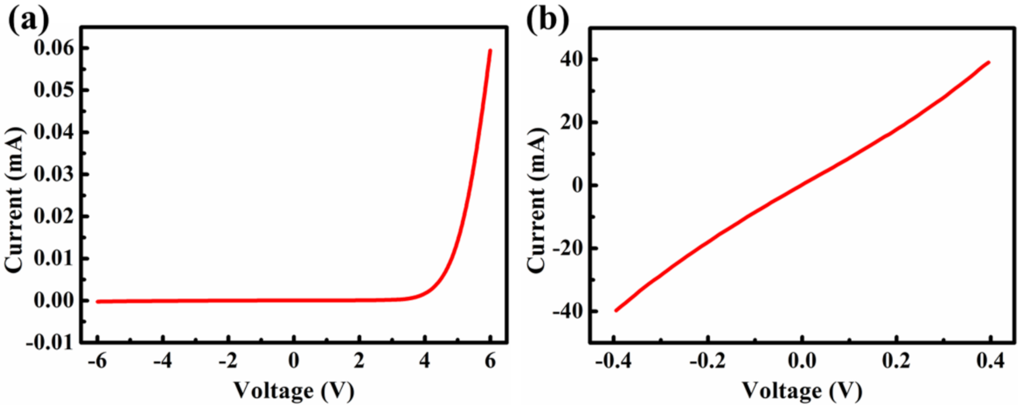

Figure S6. *I-V* curves of (a) Nb_2O_5 (N-400) and (b) Nb_4N_5 (N-700) nanochannels attached on Nb substrates.

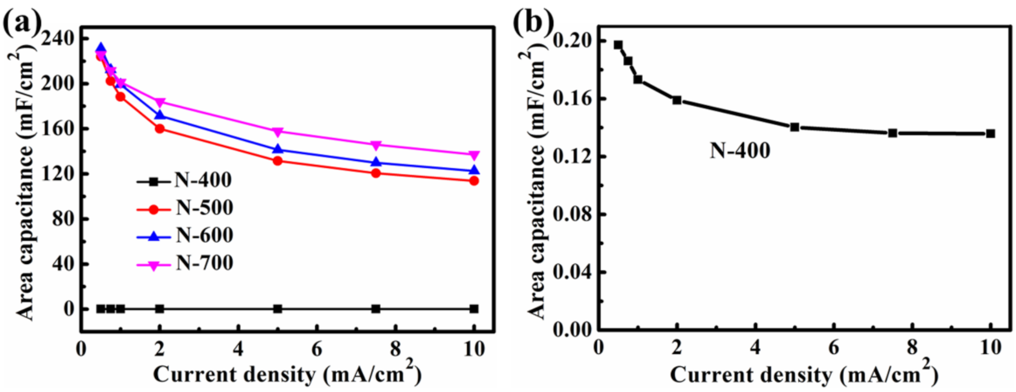

Figure S7. (a) Areal capacitance as a function of current densities of four samples nitrided at different temperature and (b) the enlarged capacitance plot for N-400

sample.

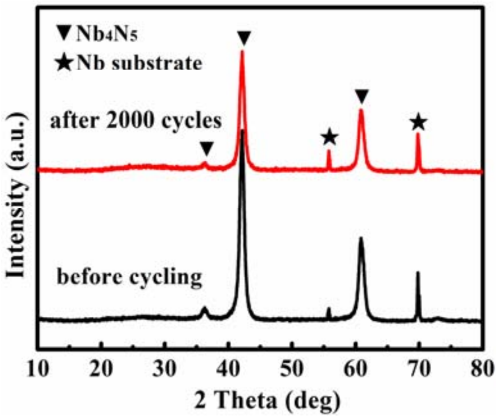

Figure S8. XRD patterns of Nb_4N_5 nanochannels measured before and after cycling.

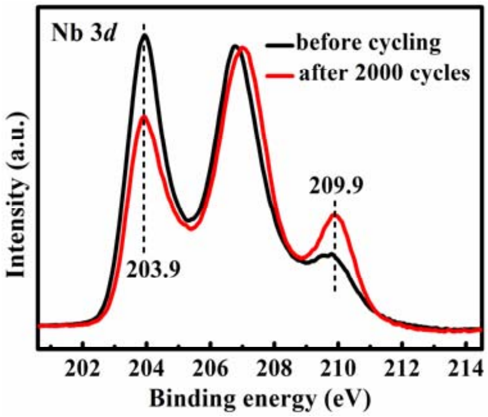

Figure S9. Nb 3*d* XPS spectra of Nb_4N_5 nanochannels measured before and after cycling.

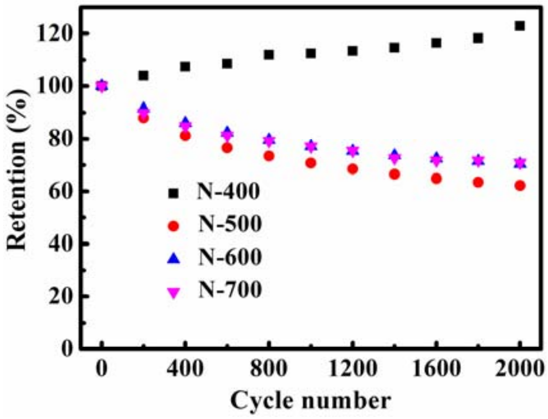

Figure S10. Cycling performance of four samples.

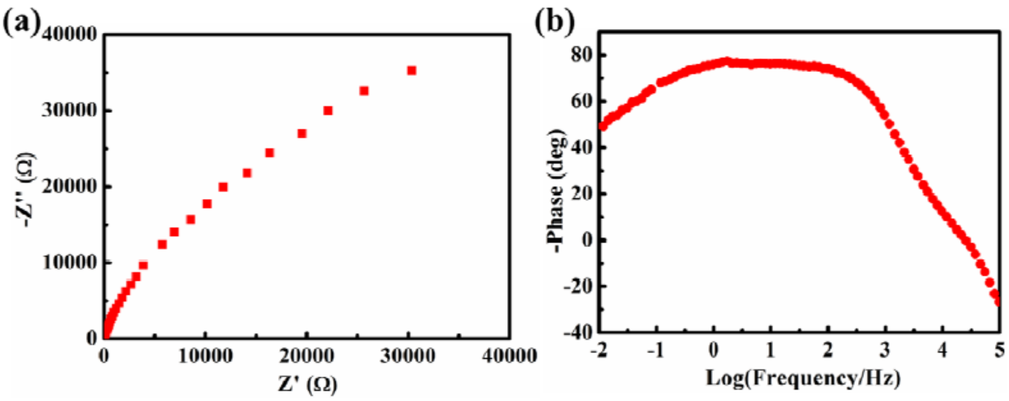

Figure S11. (a) Nyquist plot and (b) Bode phase angle plot of N-400 (Nb_2O_5) sample.

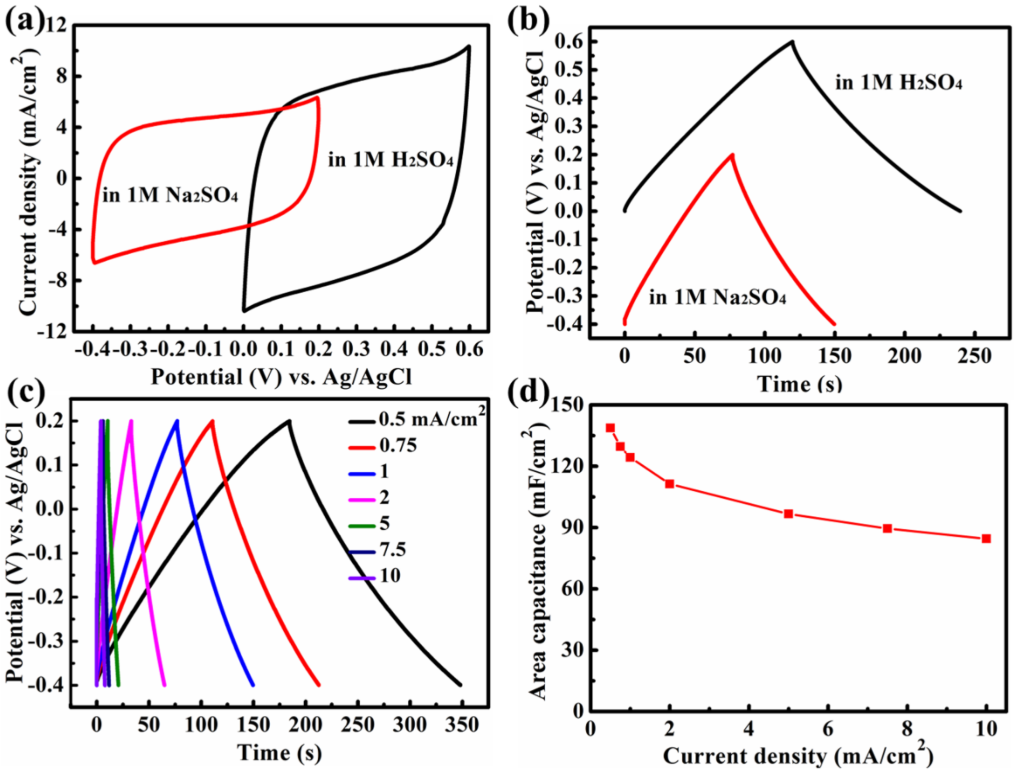

Figure S12. Comparisons of CV curves (a) at 50 mV s^{-1} and GCD curves (b) at 1 mA cm^{-2} of Nb_4N_5 nanochannels electrode measured in 1 M H_2SO_4 and 1 M Na_2SO_4 electrolyte respectively. (c) GCD curves at different current densities and corresponding areal capacitances (d) of Nb_4N_5 nanochannels measured in 1 M Na_2SO_4 electrolyte.

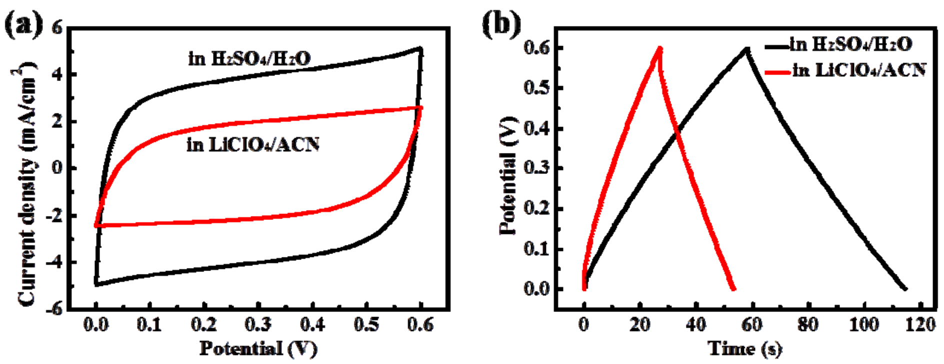

Figure S13. Comparisons of CV curves (a) at 50 mV s^{-1} and GCD curves (b) at 1 mA cm^{-2} of Nb_4N_5 nanochannels two-electrode system measured in 1 M H_2SO_4 and 1 M $LiClO_4$/acetonitrile anhydrous electrolyte.

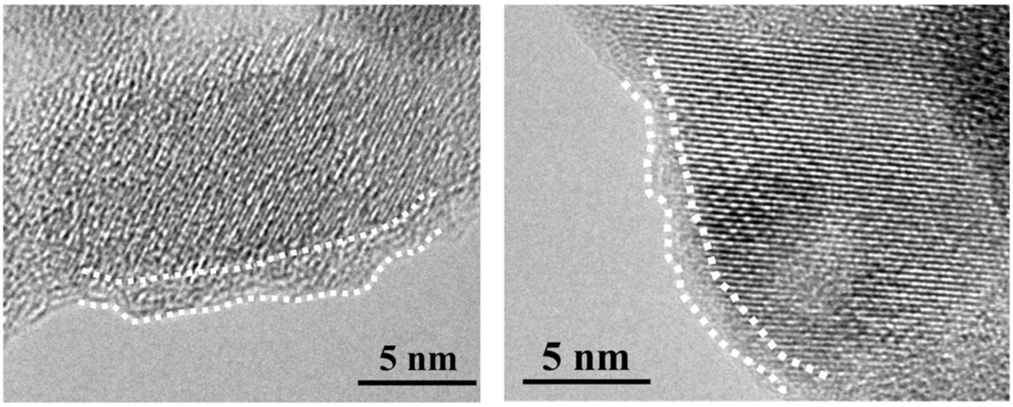

Figure S14. HR-TEM images of N-doped carbon coating Nb_4N_5 nanoparticles.

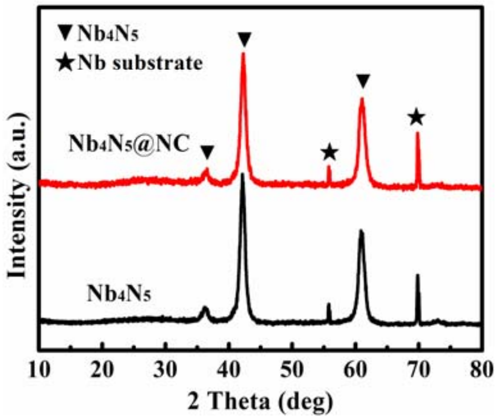

Figure S15. XRD patterns of Nb_4N_5 nanochannels with and without carbon coating.

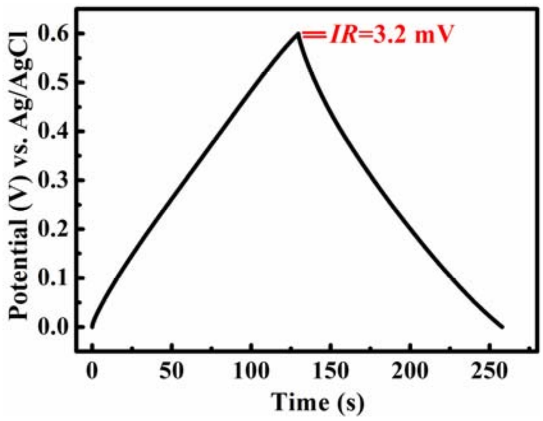

Figure S16. GCD curve of Nb_4N_5@NC nanochannels at 1 mA cm^{-2}.

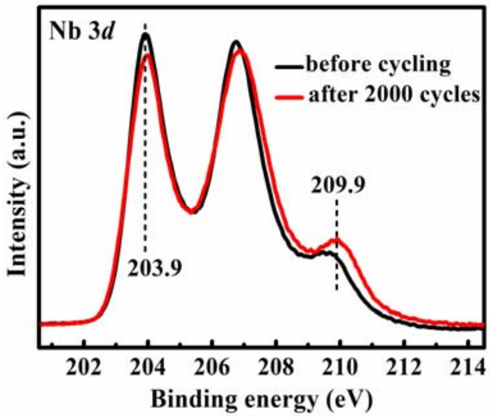

Figure S17. Nb 3*d* XPS spectra of Nb_4N_5@NC nanochannels measured before and after cycling.

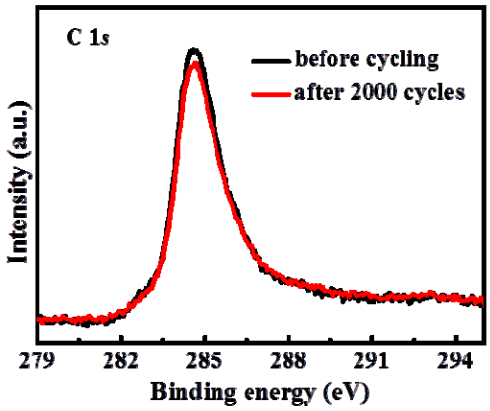

Figure S18. C 1*s* XPS spectra of Nb_4N_5@NC nanochannels measured before and after cycling.

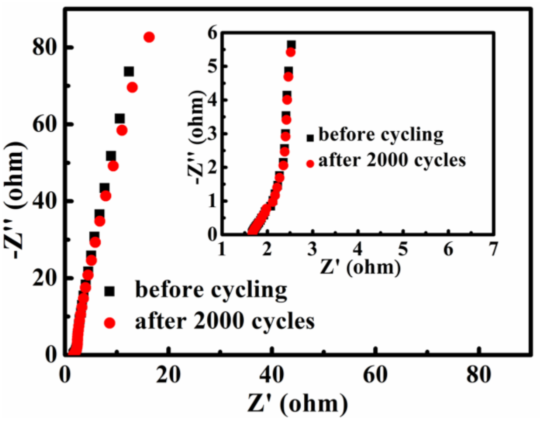

Figure S19. Nyquist plots of Nb_4N_5@NC nanochannels measured before and after cycling.

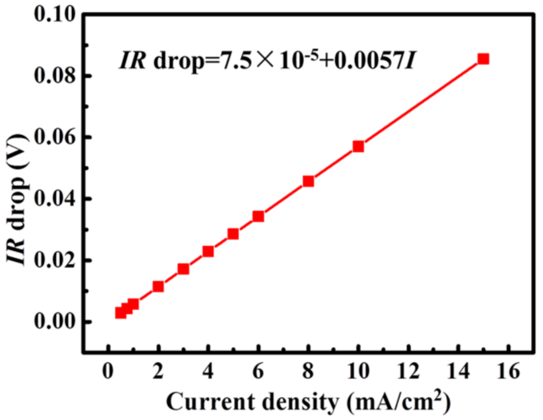

Figure S20. *IR* drop of Nb_4N_5@NC nanochannels symmetric cell vs. different discharge current densities.
